# What's beyond the core? Database coverage in qualitative information retrieval

**DOI:** 10.5195/jmla.2025.1591

**Published:** 2025-01-14

**Authors:** Jennifer Horton, David Kaunelis, Danielle Rabb, Andrea Smith

**Affiliations:** 1 jhorton@cap.org, Medical Librarian, College of American Pathologists, Northfield, Illinois, United States; 2 David.Kaunelis@cda-amc.ca, Methods Specialist, CDA-AMC (Canada's Drug Agency), Ottawa, Ontario, Canada; 3 Danielle.Rabb@cda-amc.ca, Director-Research Information Services, CDA-AMC (Canada's Drug Agency), Ottawa, Ontario, Canada; 4 Andrea.Smith@cda-amc.ca, Lead-Strategic Projects, CDA-AMC (Canada's Drug Agency), Ottawa, Ontario, Canada

**Keywords:** Informative retrieval, Qualitative research, Evidence synthesis, Database selection

## Abstract

**Objective:**

This study investigates the effectiveness of bibliographic databases to retrieve qualitative studies for use in systematic and rapid reviews in Health Technology Assessment (HTA) research. Qualitative research is becoming more prevalent in reviews and health technology assessment, but standardized search methodologies—particularly regarding database selection—are still in development.

**Methods:**

To determine how commonly used databases (MEDLINE, CINAHL, PsycINFO, Scopus, and Web of Science) perform, a comprehensive list of relevant journal titles was compiled using InCites Journal Citation Reports and validated by qualitative researchers at Canada's Drug Agency (formerly CADTH). This list was used to evaluate the qualitative holdings of each database, by calculating the percentage of total titles held in each database, as well as the number of unique titles per database.

**Results:**

While publications on qualitative search methodology generally recommend subject-specific health databases including MEDLINE, CINAHL, and PsycINFO, this study found that multidisciplinary citation indexes Scopus and Web of Science Core Collection not only had the highest percentages of total titles held, but also a higher number of unique titles.

**Conclusions:**

These indexes have potential utility in qualitative search strategies, if only for supplementing other database searches with unique records. This potential was investigated via tests on qualitative rapid review search strategies translated to Scopus to determine how the index may contribute relevant literature.

## INTRODUCTION

Qualitative evidence synthesis approaches are becoming more prevalent in health technology assessments (HTAs). Studies which employ qualitative research methods are useful when considering patient experience and preferences as well as the observations of clinical experts. Qualitative evidence synthesis (QES) is a set of methodologies used to conduct systematic evidence synthesis of primary qualitative research [1, 2]. In HTA, an evidence-based field where study design, results reporting, and review protocols are standardized, researchers and reviewers may struggle with including qualitative perspectives—which by their very nature, must be analyzed, synthesized, and critically appraised differently than clinical or economic information typically addressed in health technology assessments [3]. Though the methodological differences between reviews of effectiveness or cost-effectiveness and qualitative reviews may seem at odds with each other, more recent literature takes a reconciliatory approach, such as Booth's 2018 article on the “dual heritage” of QES [3]. Here, Booth argues that QES draws on methodologies from primary qualitative research as well as knowledge synthesis of clinical primary information, thus allowing more opportunity to utilize a variety of methodological approaches for analysis.

This study focuses on the bibliographic databases that can be used to retrieve qualitative research in the context of rapid reviews. Such reviews are carried out in a shorter time frame than systematic or scoping qualitative reviews and are often conducted in relation to a specific decision-making need and often answer more focused and narrower research questions [4, 5]. The characteristics of rapid QES inform information retrieval methodology which includes less exhaustive search strategies and a focus on a manageable number of results for an expedited timeline. Much has been written in the past ten years on how to retrieve studies for use in systematic and rapid QES, and while there is no single standard method, common practices can be pieced together [2, 6–13], which share at least three essential components—which databases to search, which search filters (if any) to employ, and how to screen or select relevant sources. This study focuses on database selection.

Just as in quantitative study searches, the databases selected have a considerable impact on the yield and relevance of qualitative results retrieved [14, 15]. Yet, prominent resources on conducting qualitative systematic reviews—such as the Joanna Briggs Institute Reviewer's Manual and the Cochrane Handbook for Systematic Reviews—do not recommend specific databases^*^ [8, 16, 17]. The Centre for Reviews and Dissemination's Guidance for Undertaking Reviews in Health Care points to recently added qualitative subject headings in MEDLINE and CINAHL but does not make an explicit recommendation to use these databases [18].

Recent literature takes up the task from which a list of databases can be compiled. The two most mentioned are MEDLINE and the Cumulative Index to Nursing and Allied Health Literature (CINAHL) [6, 11]. MEDLINE provides the most comprehensive collection of health science research and is used heavily in both quantitative and qualitative searches [2, 14]. CINAHL contains a higher percentage of qualitative research than MEDLINE, with 4% to 5% compared to 1% of total database holdings [11]. Embase and PsycINFO are also recommended. While holding unique results, Embase was employed less often in literature on qualitative searches. According to a study by Subirana et al and cited by Booth in 2016, Embase retrieved minimal unique results [2, 19]. Additionally, Frandsen et al verifies these findings and in their recommendations for database selection with maximum recall, Embase is not included in any combination [7]. Due to the authors' focus on rapid QES which necessitates a compromise on number of databases searched and yield of results that are feasible to screen in shorter amounts of time, Embase will not be included in our analysis [14]. Like CINAHL, the subject-specific nature of PsycINFO limits its utility. Subject-specificity is not always a limitation, however, an HTA organization most likely would not consider subscriptions to subject specific bibliographic databases to be particularly cost-effective considering the financial costs per use. The last databases on this list are the multidisciplinary citation indexes Scopus and Web of Science Core Collection (WoS). Until recently, as discussed more fully in Frandsen et al's 2019 article, these indexes had not been focused on or utilized as frequently as the other databases mentioned [7, 14].

The core set of databases used for other health-based systematic reviews—MEDLINE, CINAHL, and PsycINFO—is perhaps the most logical place to start searching due to their subject-specific nature, and these databases are the most likely to be already available to researchers, particularly in the HTA context. MEDLINE and CINAHL contain qualitative subject headings, and while indexing is strong for qualitative information in CINAHL, researchers will often also employ qualitative study filters to retrieve the most relevant results.

Database selection is especially pertinent in rapid QES to ensure that the retrieval of as many relevant studies is possible and feasible, focusing on a breadth and richness of differing perspectives on the same question [2, 20]. As discussed previously, recommendations for a core set of databases to search are sparse. This study aims to investigate the utility of specific databases and citation indexes to identify and balance the most—and most relevant—qualitative primary studies that are realistically manageable within the context of rapid reviews. This study can inform the selection of a core set of databases that will allow researchers to maximize the number of unique results and avoid searching more resources with fewer returns.

There is a case to be made for resources like Scopus and WoS to be included in a core set of databases to search for QES. Frandsen et al 2019 article gives strong evidence for use of Scopus, but this finding must be taken in context of their study. Tests run by Frandsen et al in Scopus did not exclude records also indexed in MEDLINE and Embase. This operation inflates the number of records retrieved by Scopus. It should be noted that running a search for MEDLINE and Embase records solely in Scopus is risky. The lack of hierarchical and standardized subject headings in Scopus that are available in MEDLINE (MeSH) and Embase (EMTREE) make searching these databases in Scopus less precise. This indicates that searches must still be run in other platforms such as Ovid which host MEDLINE or Embase to retrieve the best quality results for those databases. Frandsen et al also chose databases to analyze retroactively, based on those indicated in reports chosen. As a result, the percentage of studies retrieved by each database is again inflated, as these were the only databases searched for the reports in the first place. Frandsen's findings on Scopus nevertheless raise important questions regarding multidisciplinary citation indexes, which this present study further explores in its latter part through test searches [7].

Given that database selection is key to ensuring breadth in QES, this study aims to evaluate databases based on their holdings of qualitative information. These findings will help clarify which databases are useful and efficient when developing search strategies for QES, particularly within the context of HTA wherein researchers or information specialists may not have access to as many databases as are available at a research-intensive university. The second part of this study explores multidisciplinary citation indexes to determine how useful they may be at retrieving qualitative information in practice.

## METHODS

This study takes a modified approach to database evaluation by comparing holdings of a predetermined list of relevant journal titles. Previous studies on literature mapping for various disciplines employ similar methods to comprehensively analyze core journals to extract pertinent titles for disciplines such as social work and physical therapy [21, 22]. Similar practices are used to compare subject holdings across databases [23–25]. In the case of qualitative research related to the health sciences, a disciplinary mapping of the literature is more complex, as these studies can appear across a variety of discipline-specific publications. For this reason, the authors did not adhere to the literature mapping protocol laid out by the Medical Library Association [26]. Instead, we assessed the selected databases using a set of relevant journals to determine coverage of the topic area. After assessing the holdings of different databases, the authors sought to explore the performance of multidisciplinary citation indexes to retrieve qualitative studies. We then conducted a series of tests in Scopus to determine how searching this multidisciplinary index contributed to the overall results of a series of qualitative rapid reviews conducted by Canada's Drug Agency (formerly CADTH), a Canadian HTA agency.

The first part of the study began by compiling a list of journal titles based on a shortlist of frequently consulted titles from qualitative researchers at Canada's Drug Agency (see supplementary materials). The titles on this shortlist were searched in Clarivate Analytics InCites Journal Citation Reports (JCR) to determine which subject categories they fell into. All journal titles in the following categories were exported for analysis—Anthropology; Cultural Studies; Health Policy & Services; Social Sciences; Biomedical; and Social Issues. Categories related to health sciences but lacking a social science disciplinary aspect were excluded, as these journals are the focus of the core set of health science databases. A total of 286 titles were exported from JCR into a spreadsheet for analysis. This set was then sent to the qualitative team at Canada's Drug Agency for validation, supplementing, and secondary screening of the titles for relevancy which brought the final list to 191 titles. Using this list, the authors consulted Ulrich's Web to determine where each title was indexed. Attention was paid specifically to commonly used databases for HTA—including MEDLINE, CINAHL, PsycINFO—as well as multidisciplinary databases Scopus and WoS. Since the set of journal titles came from JCR, which draws on information from WoS and its holdings, the authors' study results on WoS are skewed. However, by assessing other databases, JCR and WoS holdings are externally validated, and the set still provides a comprehensive set with which to test these other databases. Additionally, starting with WoS holdings allowed the authors to utilize a large set of titles that is interdisciplinary, geographically diverse, and has a minimum of predatory titles. This set was also just a starting point to present to qualitative researchers to validate externally. It should also be noted here that holdings in Ulrich's Web were recorded in terms of presence or no presence and did not consider date ranges of these holdings in each database.

In addition to evaluating databases based on journals indexed, a second component of the study goes further in testing to determine how Scopus performed and contributed to previously run searches for nine published qualitative rapid reviews [27–35]. Scopus was chosen over WoS because it is the multidisciplinary databases subscribed to by the authors, and because the test set of journal titles came from JCR, a Clarivate product informed by the holdings of WoS. The authors chose nine qualitative rapid reviews to assess due to readily available information such as comprehensive search method documentation and existing EndNote libraries, which were easily accessed in-house. Search strategies from the nine rapid reviews were translated into Scopus and combined with a translation of the Canada's Drug Agency qualitative study filter <https://searchfilters.cadth.ca>. Search strings were directly translated wherever possible, with the exception of MeSH headings. If heading words or phrases were not also covered in title and abstract queries, they were added. Scopus has a very general controlled vocabulary based on journal subject categories, with headings like Social Science and Medicine which are too vague to include in a search. Though Scopus will list MeSH and EMTREE headings for articles pulled from MEDLINE and Embase, users cannot search for these terms as controlled vocabulary, only as keywords. Once the searches were translated, they were run in Scopus, with MEDLINE and Embase results excluded. These database results were excluded from Scopus searches so that the authors could evaluate Scopus on its own. Also as previously discussed, it is not generally good practice to search MEDLINE or Embase within Scopus for reviews due to less sophisticated search functionality offered on Scopus versus other platforms. Date and language limits were also applied when applicable. Search results were exported to EndNote and compared with existing libraries of literature search results for each rapid review. The authors manually deduplicated in EndNote to ensure that all Scopus results were unique. All unique Scopus results were then screened by one author who is an experienced qualitative researcher. In the first level of screening, titles and abstracts were reviewed and potentially relevant full-text articles were retrieved and assessed for inclusion. The final selection of articles was based on the inclusion criteria in the published rapid reviews.

## RESULTS

### Database Assessment via Core Journal Holdings

The set of 191 journal titles was compiled from JCR and validated by qualitative researchers at Canada's Drug Agency. This list was analyzed to determine where each title is indexed, using Ulrich's Web. Information collected from Ulrich's Web on where each journal title is indexed was further analyzed to determine the percentage of titles covered in each database, as well as the percentage of titles unique to each database. Not all 191 journals on the list were indexed in the databases studied, as indicated in Table 1. Multidisciplinary databases have the highest percentage of total holdings, with WoS at 91% and Scopus at 82%. MEDLINE had the second highest percentage of total holdings, followed by more subject-specific databases CINAHL (47%) and PsycINFO (38%).

**Table 1:** Percentage of Journal Titles per Database ^*^Web of Science Core Collection contains Science Citation Index Expanded, Social Sciences Citation Index, Arts & Humanities Citation Index, and Emerging Sources Citation Index

Database	Ratio of Total Titles	Percentage of Total Titles	Ratio of Unique Titles	Percentage of Unique Titles
MEDLINE	98/191	51%	0/191	0%
PsycINFO	73/191	38%	0/191	0%
CINAHL	91/191	48%	0/191	0%
Scopus	157/191	82%	1/191	0.5%
Web of Science^*^	175/191	92%	6/191	3%
Not indexed	13/191	7%	13/191	7%

While these results do indicate that both Scopus and WoS retrieve unique results, it is unlikely that a search approach would employ both multidisciplinary databases. There is a considerable amount of overlap between the two, which skews the percentage of unique titles for each in Table 1. More calculations were done to determine how many unique results each multidisciplinary database would yield compared to the core set of health science databases when the other was excluded from the data set (Table 2).

**Table 2:** Percentage of Unique Titles (with exclusions)

Database	Ratio of Unique Titles (with exclusions)	Percentage of Unique Titles (with exclusions)
Scopus (excluding Web of Science)	49/191	26%
Web of Science (excluding Scopus)	55/191	29%

This set illustrates a higher percentage of unique holdings when compared only with core and subject-specific health databases, indicating a considerable possible benefit for searching a multidisciplinary database in addition to common HTA resources.

### Running Searches: Scopus Assessment

To better understand the potential benefits of employing a multidisciplinary database, searches from previous CADTH qualitative rapid reviews were translated and run in Scopus to determine the number of unique results for each (Table 3). From a purely quantitative perspective, Scopus retrieves a significant number of unique results, which have the potential to facilitate the breadth of perspectives that is important in QES. Adding more results, however, does not necessarily lead to additional relevant studies. To further assess these results, qualitative researchers at Canada's Drug Agency screened citations from Scopus for inclusion for each rapid review topic. Exact results are included below (Table 4).

**Table 3:** Scopus Citation Comparison on CADTH Rapid Qualitative Reviews

Qualitative Rapid Review	Existing EndNote Records	Original Databases Searched	New Scopus Results	Percentage of New Results
Engaging with History Taking for Adverse Childhood Experiences in Care: A Rapid Qualitative Review [27]	1596	MEDLINE CINAHL PsycINFO	300	19%
Prescription Drug Monitoring Programs: A Rapid Qualitative Review [28]	89	PubMed	6	7%
Gene Expression Profiling Tests for Breast Cancer: A Rapid Qualitative Review [29]	181	MEDLINE CINAHL	24	13%
Rural Breast Cancer Surgery Programs: A Rapid Qualitative Review [30]	443	MEDLINE CINAHL	161	36%
Prostatectomy for People with Prostate Cancer: A Rapid Qualitative Review [31]	839	MEDLINE CINAHL	15	2%
Biopsy for Adults with Suspected Skin Cancer: A Rapid Qualitative Review [32]	602	MEDLINE CINAHL	10	2%
Screening and Diagnostic Services for People at Risk for Breast Cancer: A Rapid Qualitative Review [33]	995	PubMed Cochrane	54	5%
Experiences with and Expectations of Robotic Surgical Systems: A Rapid Qualitative Review [34]	1031	MEDLINE PsycINFO Scopus	283	27%
Point-of-Care Testing of International Normalized Ratios for People on Oral Anticoagulants: A Rapid Qualitative Review [35]	426	MEDLINE Embase Scopus	65	15%

**Table 4:** Relevance of Scopus Results in qualitative rapid reviews

Qualitative Rapid Review	Toted Results^*^	Included Studies^*^	Percentage of Studies Included	Total Scopus Results	Scopus Results Selected for Inclusion	Percentage of Studies Included (Scopus)
Engaging with History Taking for Adverse Childhood Experiences in Care: A Rapid Qualitative Review [27]	1596	6	0.38%	300	1	0.33%
Prescription Drug Monitoring Programs: A Rapid Qualitative Review [28]	89	18	20.22%	6	0	0%
Gene Expression Profiling Tests for Breast Cancer: A Rapid Qualitative Review [29]	181	11	6.08%	24	0	0%
Rural Breast Cancer Surgery Programs: A Rapid Qualitative Review [30]	443	12	2.71%	161	0	0%
Prostatectomy for People with Prostate Cancer: A Rapid Qualitative Review [31]	854	38	4.45%	15	0	0%
Biopsy for Adults with Suspected Skin Cancer: A Rapid Qualitative Review [32]	612	12	1.96%	10	0	0%
Screening and Diagnostic Services for People at Risk for Breast Cancer: A Rapid Qualitative Review [33]	1049	12	1.14%	54	0	0%
Experiences with and Expectations of Robotic Surgical Systems: A Rapid Qualitative Review [34]	1031	14	1%	283	0	0%
Point-of-Care Testing of International Normalized Ratios for People on Oral Anticoagulants: A Rapid Qualitative Review [35]	426	5	1.2%	65	3	0.7%

* According to report PRISMA flowcharts

Such a small set of reports tested in Scopus can only provide a limited perspective. From this test, however, just one study was selected for inclusion from the Scopus results for the first report “Engaging with History Taking for Adverse Childhood Experiences in Care” and three selected from “Point-of-Care Testing of International Normalized Ratios for People on Oral Anticoagulants” [27, 35]. Comparing total Scopus results to included Scopus studies with that of the original report results—which searched only core health science databases—indicates that this ratio can vary depending on the research question, from as high as 20% to as little as 0.38%. Percentage of studies included between the original reports and the accompanying Scopus tests are not wildly different in most cases, which may show that the ratio of included studies to the total number of results is more question-dependent as opposed to databases-specific. As such, this small sample of reports analyzed does not represent a large breadth of research topic areas. One can imagine that in other topic areas, utilizing multidisciplinary citation indexes could be more useful.

## DISCUSSION

As observed from the first part of this study, databases CINAHL and PsycINFO did not include any unique holdings based on the list of journals searched for. However, their controlled vocabulary and indexing are unique, and it is likely one would retrieve unique citations that may be relevant for QES. Thus, a search in CINAHL can retrieve unique results. This validates the methodological practice of searching multiple databases that have similar holdings, as results may differ based on the search strategy which may include different subject headings, holding completeness of certain titles (date ranges of title held), and search functionality specificities.

Scopus and Web of Science have a high number of unique journal titles that would not be searched at all if only adhering to core health science databases. It also important to note that Scopus searches tested do not add significantly to the original report results—meaning that the total number of results would still be a reasonable amount for a reviewer to screen. Scopus is proven here for retrieval of unique results, but more tests must be done to assess the quality of results retrieved by a multidisciplinary citation index such as Scopus, and changes to search strategy (aside from a direct translation) may be necessary.

This research is subject to several limitations. The authors' decision to exclude Embase from study was informed by literature on the topic, but there is no consensus on whether searching Embase for qualitative literature is beneficial. Thus, this study cannot recommend or discourage use of Embase, and further research must be conducted to evaluate this database.

Journal titles were chosen based on subjects in JCR and supplemented by qualitative researchers at Canada's Drug Agency, but the list used is not an exhaustive one. JCR, the product used to compile an initial list of journal titles, solely includes holdings of WoS and does not take into account additional holdings of Scopus. This resulted in Scopus's holdings being underreported by the authors. Further research must be done using other journal lists to better determine the breadth of qualitative titles in Scopus. The authors' decision to use Ulrich's Web to evaluate database holdings did not account for date ranges of each journal held in each database. This may imply complete journal coverage in a given database, but actual holdings were not verified in this study. A more accurate approach may have been to have searched the journal title list of each database, however additional research may be conducted to more accurately verify database holdings. Scopus test cases illustrate that implementation is most important, but such concerns as journal date coverage is built into common practice for systematic searching. Further investigation into date range discrepancies between databases could be conducted, but it was out of scope for this study.

In addition to being limited to the researchers' experience with and familiarity of journal titles, it is difficult to determine exactly where qualitative research is being published, especially because more and more qualitative studies are being included in otherwise clinical publications. Qualitative searching and the volume of literature retrieved are heavily dependent on the research topic and question, and while this study's sample of nine qualitative rapid responses provides some insight into how multidisciplinary databases contribute to a search, it is far from being comprehensive given the limited number of research topics covered by these reviews.

Additionally, database selection is just one component to information retrieval. Other factors such as search strategy and filter selection have an impact on the volume and relevancy of literature results. This study is also biased towards the needs—and resources—of the organization at which it was conducted. The research practice and goals of Canada's Drug Agency are characteristic of the HTA field but its individual mission and the resources available inform the purpose and limitations of our study.

This study focused on rapid reviews specifically and did not address other more comprehensive forms of knowledge synthesis. Traditional systematic or scoping reviews have different methodological requirements and may necessitate use of more databases than those studied here. Testing database search capabilities in those contexts therefore may produce different results than the rapid reviews studied here. Research questions and search strategies for rapid reviews are typically more focused than those for a systematic review, and this focus may have limited the number of results retrieved in the authors' test of Scopus. In other words, a broader search question may yield more potentially relevant results.

Lastly, the decision to directly translate search strings from MEDLINE to Scopus for the evaluation of a multidisciplinary citation index influenced the number and quality of results retrieved. MeSH terms from the original searches could not be searched or adequately translated due to the broad nature of Scopus's Subject Area controlled vocabulary.

The findings presented here further our understanding of the utility of various databases in QES and simultaneously raise more questions related to qualitative search methodology. Considering the percentage of unique journal titles indexed in Scopus and WoS, what place do these resources have in regard to QES information retrieval methods? If multidisciplinary citation indexes were included along with the core health science databases, in what ways would search strategies need to change to yield results of the best quality—and of reasonable quantity? Might Scopus journal subject categories be employed to exclude out-of-scope disciplines such as chemistry, engineering, and physics?

These questions help determine next steps for further study. To gain more accurate insights into Scopus performance in QES, information specialists at Canada's Drug Agency will continue to search the database on a trial, case-by-case basis. Information specialists working on projects with a qualitative focus or component (such as rapid and systematic reviews) will search Scopus in addition to core health science databases. Number of Scopus results retrieved, and studies to be included will be documented in a similar manner to the tests done on previous rapid reviews. In addition, there will be opportunities to consult with the information specialists and qualitative reviewers on their reflections and lessons learned to adjust and improve Scopus search practice.

## CONCLUSION

Along with the set of core health science databases (MEDLINE/PubMed, PsycINFO, CINAHL), it can be beneficial to include Scopus or Web of Science as a supplemental source of qualitative information. These multidisciplinary indexes contain unique journals which publish relevant study types—studies which may otherwise be missed if only searching core health science databases. At the same time, the authors acknowledge the potential limitations of searching in Scopus or Web of Science. Search functionality in these indexes is not as complex or controlled as searching in health science databases, particularly due to the lack of an adequate controlled vocabulary. These databases are also costly to license, which may make them inaccessible to HTA agencies or other organizations. One must also note that the yield of relevant information from Scopus or Web of Science is question-specific. Though the ideal standard for evidence syntheses is to locate and consider all possible sources of information, research teams must contend with how they can at once be most efficient and most thorough, balancing precision with sensitivity given resource constraints and shortened time frames. Further research should be conducted to determine just how useful the addition of a multidisciplinary index like Scopus or Web of Science may be to rapid qualitative evidence synthesis projects—as well as search or translation strategies which best fit these indexes—though this study provides evidence that such indexes show promise in the field of QES.

## Data Availability

Data and search strategies associated with this article are available in the Open Science Framework at https://osf.io/dj7gf/
